# *PuCRZ1*, an C2H2 transcription factor from *Polyporus umbellatus*, positively regulates mycelium response to osmotic stress

**DOI:** 10.3389/fmicb.2023.1131605

**Published:** 2023-04-06

**Authors:** Pengjie Han, Zhongyi Hua, Yuyang Zhao, Luqi Huang, Yuan Yuan

**Affiliations:** ^1^School of Pharmaceutical Sciences, Peking University, Beijing, China; ^2^State Key Laboratory of Dao-di Herbs, National Resource Center for Chinese Materia Medica, China Academy of Chinese Medical Sciences, Beijing, China

**Keywords:** *Polyporus umbellatus*, CRZ1 transcription factor, DAP-seq, stress tolerance, Ca^2+^ pathways

## Abstract

*Polyporus umbellatus* is an edible and medicinal mushroom with the capacity to produce sclerotia. However, the mechanism of *P. umbellatus* sclerotia formation is unclear. CRZ1 is a C2H2 family transcription factor involved in the Ca^2+^-calcineurin signaling pathway, which has the function of regulating sclerotia formation, maintaining ion homeostasis, and responding to stress. In this study, we identified 28 C2H2 transcription factors in *P. umbellatus* genome, 13 of which are differentially expressed between mycelium and sclerotia, including *PuCRZ1*. Combining DNA affinity purification and sequencing (DAP-seq) and quantitative real-time PCR (qRT-PCR), three genes (*PuG10*, *PuG11*, *PuG12*) were identified as putative *PuCRZ1* target genes containing a putative binding motif (GTGGCG) within their promoter. Yeast single hybridization (Y1H) and EMSA further confirmed that *PuCRZ1* can bind to the promoter region of *PuG10*, *PuG11*, and *PuG12*. PuCRZ1 gene could reduce the sensitivity of NaCl in yeast cells. Furthermore, overexpression of the PuCRZ1 target gene, especially the FVLY domain containing gene *PuG11*, could improve the mycelia growth rate and mannitol tolerance in *P. umbellatus*. These results demonstrate that *PuCRZ1* in the Ca^2+^-calcineurin signaling pathway plays an important role in mycelia growth, as well as osmotic stress tolerance.

## 1. Introduction

*Polyporus umbellatus* is a well-known edible and medicinal mushroom. As a kind of traditional Chinese medicine, *P. umbellatus* sclerotia has a medicinal history of 2,500 years in China (Xing et al., 2015; Chinese Pharmacopoeia Commission, 2020). In recent years, the polysaccharides extracted from the sclerotia of *P. umbellatus* have been shown to have anti-cancer activity, immunomodulatory activity, anti-oxidant activity, anti-inflammatory activity and renoprotective activity (Li et al., 2019b; Liu et al., 2021). The sclerotia of fungi is a dormant structure for some fungi to resist adverse environment (Xing et al., 2013), and is also a necessary stage for the formation of fruiting body (Smith et al., 2015; Liu et al., 2018). The sclerotia of fungi are composed of hyphae that gradually develops into a sclerotium after forming a hyphal knot (Bandara et al., 2015). The main structure of sclerotium consists of an outer layer formed by closely arranged cells with thickened cell wall and an inner tissue formed by loosely arranged vegetative cells (Bandara et al., 2015). Due to the slow development of *P. umbellatus* sclerotia and its relatively stringent requirements for environmental conditions, the molecular mechanism of sclerotia formation is still unclear.

The calcium signaling pathway plays an important role in sclerotia formation and fungal resistance. The Ca^2+^-calcineurin signaling pathway is a widely conserved calcium signaling pathway in animals and fungi, which regulates life activities such as metal ion homeostasis, cell cycle, cell growth and differentiation, and cell wall synthesis (Berridge et al., 2003; Yang et al., 2022). The CaN-CRZ1 signaling cascade in fungal cells can be activated by different external stimuli, such as high/low temperature, hypertonicity, alkalinity, oxidative stress, and others (Yang et al., 2022). In fungal cells, Calcineurin responsive Zing finger transcription factor (CRZ1) was first identified in *Saccharomyces cerevisiae* (Stathopoulos and Cyert, 1997). Subsequently, homologs of CRZ1 gene were identified in different species (Gupta et al., 2022; Yang et al., 2022).

All CRZ1 homologs have one or more signature C2H2 zinc finger domains, and their functions and regulatory genes differ among different species (Gupta et al., 2022; Yang et al., 2022). Crz1- regulated genes are involved in sclerotia formation in *Verticillium dahliae* (Xiong et al., 2015) and *Valsa pyri* (He et al., 2016), impaired mycelium growth and abnormal branching in *Botrytis cinerea* (Schumacher et al., 2008), spores and conidia produced in *B. cinerea*, *Metarhizium acridum* (Chen et al., 2017) and *Fusarium graminearum* (Chen et al., 2019), morphological transformation, irregular plasma membrane structure and abnormal organelles in *Candida lusitaniae* (Zhang et al., 2012).

The CRZ1 deletion mutants showed different responses to ion stress in different fungi (Yang et al., 2022), i.e., sensitive to Na^+^, Li^+^, Mn^2+^, and Ca^2+^ in *B. cinerea*, to only Mn^2+^ in *Aspergillus fumigatus*, and insensitive to Na^+^, Li^+^, and Mn^2+^ in *Magnaporthe grisea* (Schumacher et al., 2008; Soriani et al., 2008; Zhang et al., 2009). Although CRZ1 and its homologs have been reported in stress response, biosynthesis of secondary metabolites and fungal virulence, there are few studies on the CRZ1 gene in basidiomycetes. The regulatory role of CRZ1 in ganoderic acid biosynthesis has only been investigated in *Ganoderma lucidum* (Li and Zhong, 2020). Previous studies have reported that environmental factors (oxidation, temperature, medium) affect the formation of *P. umbellatus* sclerotia (Xing et al., 2013, 2015, 2021; Bandara et al., 2015). Here, we further investigated the function of the transcription factor CRZ1 in *P. umbellatus* and whether it is associated with resistance and sclerotia formation like other fungi. In this study, we used qRT-PCR, DAP-seq, yeast one-hybrid (Y1H) assay, EMSA and *Agrobacterium*-mediated genetic transformation to analyze the expression levels of *PuCRZ1* in different stages of *P. umbellatus*, *PuCRZ1* target genes and their effects in mycelial growth of *P. umbellatus*. The results revealed that PuCRZ1 in *P. umbellatus* could regulate genes containing the GTGGCG motif in their promoter regions and increase resistance to osmotic stress by regulating target genes encoding the FLVY-domain-containing proteins.

## 2. Materials and methods

### 2.1. Materials

Sclerotia samples of *P. umbellatus* were collected from Ningshan, Shaanxi Province, China (33°309″ N, 108°828″E). The *P. umbellatus* strain (ID: 5.173) was obtained from the China Microbial Strain Collection Center, grown on potato dextrose agar (PDA) medium (Solarbio, Beijing, China) in the laboratory.

### 2.2. Identification of C2H2 gene family members in *P. umbellatus* and phylogenetic analysis

C2H2 genes encoded in the *P. umbellatus* genome were identified by two separate methods. First, hidden Markov models (HMMs) of the C2H2 domain were downloaded from the Pfam database and used to search the 10864 predicted *P. umbellatus* protein sequences. Thirty-seven genes exhibited matches. To validate the C2H2 candidates acquired using the Pfam HMMs, the 37 matching genes were analyzed using the Interpro online tools. Second, using 53 C2H2 protein sequences in yeast, 78 matching genes were identified in the *P. umbellatus* genome by BLASTP. Finally, the C2H2 candidates obtained from these two methods were integrated, yielding a total of 28 C2H2 candidates in the *P. umbellatus* genome.

### 2.3. RNA isolation and cloning full-length cDNA of *PuCRZ1*

Total RNA was extracted from mycelia using the RNA prep Pure plant kit (polysaccharides and polyphenolics-rich) (Tiangen, Beijing, China) according to the manufacturer’s instructions. First-strand cDNA was synthesized using 1.0 μg of total RNA with EasyScript^®^ One-Step gDNA Removal and cDNA Synthesis SuperMix Kit (TransGen Biotech, Beijing, China). PCR cloning of full-length cDNA of *PuCRZ1* was performed in a total volume of 50 μl containing 1 μL of cDNA product, 25 μL 2 × TransStart FastPfu PCR Super Mix and 2 μl of forward and reverse primers (10 μM) designed based on the PuCRZ1 sequence from *P. umbellatus* genome. The 5′ to 3′ primer sequences were: forward, 5′ATGTCGGACCGCATTCAACAGT-3′; and reverse, 5′-TCACGACGACTCGTCAATACCG-3′. The PCR product was gel purified and then ligated to the pEASY^®^-Blunt Cloning Vector (TransGen Biotech, Beijing, China) for DNA sequencing.

### 2.4. Subcellular localization

To determine subcellular location of the protein encoded by *PuCRZ1*, *Agrobacterium tumefaciens* GV3101 was transformed with a *PgpdA*-eGFP vector containing an in-frame fusion of *PuCRZ1* with eGFP. The constructs and empty vector were transiently transformed into *P. umbellatus* fresh mycelium, respectively. Putative transgenic fungi were selected on PDA medium containing 10 mg/L *Hygromycin B* and verified by PCR using specific primers (PJ01-F/PJ02-R, Table 1). Transgenic fungi were incubated in the dark at 25°C for 60 days. Subcellular protein localization was analyzed at 0.5 h after infiltration by DAPI (100 ng/ml) using a laser scanning microscope (ZEISS LSM 880, Germany).

**TABLE 1:** Primers in study.

Name	Sequence
Pu-CRZ-F1	ATGTCGGACCGCATTCAACAGT
Pu-CRZ-R1	TCACGACGACTCGTCAATACCG
PJ01-F	CCAGCTGCTCTTTTCTTTTC
PJ02-R	CCGAAACGCGTTTTATTCTT
PJ05-PuG10-F	ATGGACGCCGTGAAGGCCGTTGGG
PJ06-PuG10-R	CTATGGCTGAGAAGCAATCCGGTCCA
PJ11-PuG11-F	ATGGAGAATCCCCCATATGTCCCTTAC
PJ12-PuG11-R	CTAGATGTCCGTGGGTGTCCGT
PJ13-PuG12-F	ATGTCCGCCATGCGTGCGGT
PJ14-PuG12-R	CTACGATGTAGTAGACTCGCGGAGCC
PJ-21-PuG12pr-F1	Acctcgacagtccatccccgaa
PJ-22-PuG12pr-R1	gcggcagtagagggtgtcagta
PJ-25-PuG10pr-F1	cgcacggcattcaacttgaa
PJ-26-PuG10pr-R1	tagctcgcctaccaggtagt
PJ-35-PuG11pr-F1	ataataccacctgtcgggcg
PJ-36-PuG11pr-R1	actaaccgcgccttgagtac
**RT-PCR primers**	
β-Tublin-R-qpcr	CCTTCCTTGGCAACTCGACA
β-Tublin-F-qpcr	TCGTCCATACCCTCCTGTGT
PuCRZ1-F	CACCCGAACACTACCCATCTT
PuCRZ1-R	CAGCTAGGTTTTGTGGAGAAGAC
PuG10-F	GAAAAAGCCTGTCATCCGATACG
PuG10-R	TCCAATATGAGCAGTAATGGGGG
PuG11-F	GGTTGTGGCTCATACTGTTCATG
PuG11-R	CTGATGTGGGAGATGTATCAGGG
PuG12-F	TATCAACATCTCTTCTCGCCTCG
PuG12-R	GGCGACAAGATAGTAGCGGATAT

### 2.5. Transformation of *PuCRZ1* in yeast strains

The *PuCRZ1* gene was subcloned into the pYES2 vector and transformed into a competent yeast (*S. cerevisiae* BY4741). Meanwhile, the empty vector was transformed as a wild type control. Positive transformed yeast strains were selected by synthetic defined (SD) medium deficient in Ura (SD/-Ura). After obtaining the transformed yeast strains, they were cultured in SD/-Ura liquid medium to OD_600_ = 1.0, and then diluted 100, 1000, 2000, 5000, 10000 times with SD/-Ura liquid medium with D-galactose. After dilution, 2 μl of yeast was inoculated into SD/-Ura solid medium containing NaCl or CaCl_2_ with D-galactose. The plates were incubated at 30°C for 3 days.

### 2.6. Quantitative real-time PCR analysis of *PuCRZ1* and target genes in *P. umbellatus*

Total RNA was extracted from *P. umbellatus* mycelia and sclerotia using the RNAprep Pure plant kit (polysaccharides and polyphenolics-rich) (Tiangen, Beijing, China) according to the manufacturer’s instructions.

First-strand cDNA was synthesized using 1.0 μg of total RNA with EasyScript^®^ One-Step gDNA Removal and cDNA Synthesis SuperMix Kit (TransGen Biotech, Beijing, China). Tip Green qPCR SuperMix Kit (TransGen Biotech, Beijing, China) was used to conduct RT-PCR on LightCycler 480 II system (Roche Applied Science, Mannheim, Germany). Each RT-PCR mixture contained 1 μL cDNA, 1 μL primers, 5 μL Tip Green qPCR SuperMix, and 3 μL nuclease-free water. The qRT-PCR conditions were as follows: initial denaturation at 94 °C for 30 s, followed by denaturation at 94 °C for 5 s and annealing of primers at 60 °C for 20 s, and extension at 72 °C for 20 s. The samples were cooled to 60 °C and then heated to 94 °C by 40 cycles, and the melting curves were generated. Three biological replicates were established in each treatment group and β-tublin was used as an endogenous reference. Three technical replicates were analyzed for each biological sample. Table 1 and Supplementary Table 1 lists the forward and reverse primers for *PuCRZ1*, *PuG1-12* and β-tublin. The expression of the target gene was normalized to that of the reference gene (β-tublin) using the 2^–ΔΔCt^ method.

### 2.7. DNA affinity purification sequencing (DAP-seq)

DAP-seq binding assays were performed as described previously with modifications as described briefly herein (Bartlett et al., 2016; O’Malley et al., 2016).

#### 2.7.1. Nuclear isolation and genomic DNA exaction

Fresh *Polyporus umbellatus* mycelia were ground to a fine powder using liquid nitrogen. The powder was resuspended in 2 mL cold nuclear isolation buffer. The solution was then filtered through 30 μm cell strainer and centrifuged at 2000 × *g* for 4 min at 4 °C. The supernatant was discarded. The precipitate was resuspended in 100 μL PBS, and added 1 μL RNase at 37°C for 30 min, 3 μL proteinase K at 55°C for 30 min, 100 μL 2 × CTAB at 65°C for 30 min, then 200 μL of phenol/chloroform/isoamyl alcohol was added and the solution was thoroughly mixed using a vortex. The solution was centrifuged at 11000 × *g* for 10 min at room temperature. The supernatant was transferred into a new 1.5 mL eppendorf tube, and added 2.5 × volume ethanol, incubated at 20°C for 1 h and then centrifuged at 11000 × *g* for 10 min at room temperature. The supernatant was discarded, and the tube was dried at room temperature. The DNA was dissolved in 50 μL of Tris EDTA (TE) buffer.

#### 2.7.2. DAP-seq genomic DNA library preparation

Genomic DNA (gDNA, 5 μg in 130 μL TE buffer) was fragmented to an average of 200 bp using a Covaris M220 (Woburn, MA, USA) according to the manufacturer’s recommended settings. The fragmented gDNA was then purified using AMPure XP beads (Beckman Coulter, Inc., Indianapolis, IN, USA) at a DNA to beads ratio of 0.7–1.1. The beads were incubated with the gDNA for 5 min at room temperature, and placed on a magnet to immobilize the beads. The supernatant was then removed. The beads were washed twice with 200 μL of 80% ethanol and allowed to dry. Once dry, they were resuspended in 22 μL resuspension buffer, incubated at room temperature for 5 min, and placed on the magnet. The DNA-containing supernatant was then transferred to a new tube. Libraries were constructed using the NEXTFLEX Rapid DNA-Seq Kit (PerkinElmer, Inc., Austin, TX, USA) according to the manufacturer’s instructions.

#### 2.7.3. DAP-seq protein expression

The coding sequencing of *PuCRZ1* was cloned into a pFN19K HaloTag T7 SP6 Flexi expression vector. Halo-*PuCRZ1* fusion protein was expressed using the TNT SP6 Coupled Wheat Germ Extract System (Promega) following the manufacture’s specifications for expression in a 50 μL reaction with a 2 h incubation at 37°C. Expressed proteins were directly captured using Magne Halo Tag Beads (Promega).

#### 2.7.4. DAP-seq binding assay and sequencing

The protein-bound beads were incubated with 50 ng of adapter-ligated gDNA fragments on a rotator for 1 h at room temperature in 50 μL wash/bind buffer. Beads were washed three times using the same wash buffer to remove unbound DNA fragments. The HaloTag beads were resuspended in 30 μL of elution buffer and heated to 98°C for 10 min to denature the protein and release the bound DNA fragments into solution. The supernatant was transferred to a new well, and 25 μL were used in a 50 μL PCR employing the KAPA HiFi HotStart ReadyMixPCR Kit (Roche, Basel, Switzerland) for 10 cycles. PCR primers consisted of the full-length Illumina TruSeq Universal primer (5′-AATGATACGGCGACCACCGAGATCTACACTCTTTCCCTACA CGACGCTCTTCCGATCT-3′) and an Illumina TruSeq Index primer (5′-CAAGCAGAAGACGGCATACGAGATNNNNNNGT GACTGGAGTTCAGACGTGTGCTCTTCCGATCT-3′) where NNNNNN represents the 6 bp sequence index used for sample identification. The PCR product was purified and selected using AMPure XP beads (Beckman) as described above, and resuspended in 20 μL nuclease-free water. DNA concentrations were determined using a Qubit (Life Technologies, Burlington, ON, Canada). Eluted DNA fragments were sequenced on an Illumina NavoSeq. Negative control mock DAP-seq libraries were prepared without the addition of protein to the beads.

#### 2.7.5. DAP-seq data processing

Reads were mapped to the *P. umbellatus* genome sequence using BOWTIE2 (Langmead and Salzberg, 2012). Peak calling was done using Macs2 (Zhang et al., 2008).

DAP-seq peaks located within 2 kb upstream or downstream of the transcription start site (TSS) were analyzed using the Homer (Heinz et al., 2010). Gene function annotation was blasted from the following NCBI BLAST databases: NR, NT, Swissprot, and Pfam. FASTA sequences were obtained using BEDTools for motif analysis (Quinlan and Hall, 2010). Motif discovery was performed using the MEME-ChIP suite 5.0.5 (Machanick and Bailey, 2011).

### 2.8. Yeast one-hybrid (Y1H) assay

Using Gold Matchmaker™ (Clontech Laboratories, Inc., CA, USA), Y1H assays (Ji et al., 2014; Yao et al., 2020) were used to verify the interaction between PuCRZ1 and the GHGGH motif of the *PuG10*/*PuG11*/*PuG12* promoter (*proPuG10*, *proPuG11*, and *proPuG12*). The *proPuG10*, *proPuG11*, and *proPuG12* was isolated *via* a series of PCR amplifications. Table 1 shows the primer sequences used for *proPuG10*, *proPuG11*, and *proPuG12* isolation. The tandem copies of the *proPuG10*, *proPuG11*, and *proPuG12* were inserted into the pAbAi vector as bait. These bait constructs were integrated separately into the genome of the Y1HGold to generate three bait reporter strains. The minimal inhibitory concentrations of Aureobasidin A (AbA) were determined for bait using SD/–Uracil (Ura) agar plates containing 100–1000 ng/mL. *PuCRZ1* was subcloned into the pGADT7 vector as prey. The pGADT7-*PuCRZ1* construct was introduced into the bait reporter strains, with a blank pGADT7 plasmid serving as a negative control. Positive transformants were selected on SD/–Leucine (Leu) with an appropriate concentration of AbA medium. Yeast cells were grown for 3 days at 30 °C. Y1H assays were repeated three times, and representative results are shown.

### 2.9. Electrophoretic mobility shift assay (EMSA)

Electrophoretic mobility shift assay was performed as described previously (Zheng et al., 2021). The full-length cDNA of *PuCRZ1* was cloned into pET32a (+) vector (Invitrogen, CA, USA), and then transformed into the *Escherichia coli* strain BL21 (DE3) competent cell (TransGen Biotech, Beijing, China). An empty pET32a (+) vector was used as a negative control. Prokaryotic expression was performed at 16°C for 16 h with 0.5 mM isopropyl-β-d-thiogalactoside (IPTG), and then recombinant protein was purified using Ni-NTA column (TransGen Biotech, Beijing, China), and the SDS-PAGE result of purified PuCRZ1 protein in Supplementary Figure 5. Primers and probes are listed in Supplementary Table 2. Unlabeled probes were subjected to cold competition experiments. EMSA were performed using the Chemiluminescent EMSA kit (Beyotime, Shanghai, China) according to the manufacturer’s instructions. The binding reactions were performed using 1 μg of 6 × His-PuCRZ1 incubated with 7.5 nM probe in binding buffer for 30 min at room temperature. The 6 × His protein was used as a negative control. The reaction mixtures were separated in 6% native polyacrylamide gel electrophoresis (PAGE) gel. Biotin activity was detected according to the manufacturer’s instructions. The EMSAs were repeated three times, and representative results are shown.

### 2.10. Transient overexpression in *P. umbellatus*

To construct the *PgpdA:: PuCRZ1:: PgpdA*, *PgpdA:: PuG10:: PgpdA*, *PgpdA:: PuG11:: PgpdA*, and *PgpdA:: PuG12:: PgpdA*, overexpression vector, the fragment containing the *PuCRZ1*/*PuG10* /*PuG11*/*PuG12* ORF was amplified from Blunt-*PuCRZ1* vector using specific primers (CRZ1-OE-F/R) and then the *PuCRZ1* ORF was cloned into the *SmaI* sites of the pAg1-OE vector driven by the PgpdA promoter. The *PgpdA:: PuCRZ1:: PgpdA* construct was introduced into *A. tumefaciens* strain GV3101, and then transformed into WT *P. umbellatus* fresh mycelium using an *Agrobacterium*-mediated method. The blank pAg1-OE vector was introduced into WT *P. umbellatus* fresh mycelium as a control. Putative transgenic fungi were selected on PDA medium containing 10 mg/L Hygromycin B and verified by PCR and semiquantitative RT-PCR using specific primers (Table 1). WT and transgenic fungi were incubated in the dark at 25°C for 30–50 days.

Among the 17 independent transgenic lines, two independent transgenic lines (P-13 and P-17) exhibited higher abundance of *PuCRZ1* transcript and were used for Phenotype tests. Among the 6/5/8 independent transgenic lines, three/two/three independent transgenic lines (*PuG10*-P1/P5/P10, *PuG11*-P10/P11, *PuG12*-P5/P8/P9) exhibited higher abundance of *PuG10/PuG11/PuG12* transcript and were used for phenotype tests.

### 2.11. Phenotype tests of transgenic *P. umbellatus*

A fungal disc (6 mm in diameter) was placed in the center of a modified potato dextrose agar (mPDA) with NaCl (20 mM), mannitol (150 mM) or CaCl_2_ (5 mM) plate. Plates were incubated in the dark at 25°C for 30 days. The mycelial growth was measured by the diameter of the colony. Phenotype tests were repeated six times, and representative results are shown.

## 3. Results

### 3.1. Molecular characterization of a C2H2 zinc finger gene in *P. umbellatus*

**FIGURE 1:**
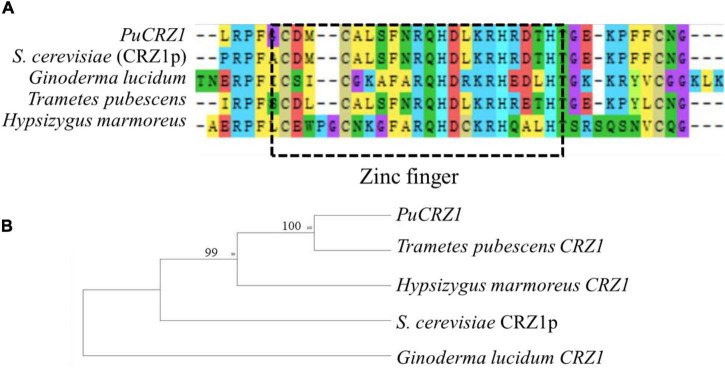
Comparison of PuCRZ1 from *P. umbellatus* with other fungal homologs. **(A)** Amino acid sequence alignment PuCrz1 of *P. umbellatus* and homologs of CRZ1 from *S. cerevisiae* (CRZ1p), *Ganoderma lucidum*, *Trametes pubescens*, and *Hypsizygus marmoreus*. The box with the dashed line indicates the conserved C2H2 motif (C2H3-type domains). **(B)** Phylogenetic analysis of *PuCZ1* and other homologs from other fungal species. The phylogenetic tree was constructed using MEGA 6.0 with full length of protein sequences and a neighbor-joining method with 1000 bootstrap replications.

In this study, we combined two different approaches, C2H2 domain search and homologous protein sequence alignment, to identify 28 C2H2 family genes in *P. umbellatus*. Of these, 13 genes were differentially expressed between mycelium and sclerotia (Table 2), including the CRZ1 homolog (*PuCRZ1*). Gene expression levels were verified using qRT-PCR (Supplementary Figure 1), and the transcript levels of *PuCRZ1* were significantly different between mycelium and sclerotia. The *PuCRZ1* gene is 801 bp in length and encodes a protein of 266 amino acids, which contains a typical C2H2 motif (C2H3-type) (Figure 1). Phylogenetic analysis of *PuCRZ1* and homologs from other fungi demonstrated that CRZ1 are relatively conserved among basidiomycetes (Figure 1). It was also found that the CRZ1 sequences in basidiomycetes were less than 300 residues, whereas those in ascomycetes were greater than 600 residues. Although the core domain of CRZ1 is conserved, the length of the amino acid sequence encoded varies greatly among species, which is why the homologous genes have different functions in different species.

**TABLE 2:** C2H2 family genes were differentially expressed in sclerotia and mycelia.

ID	Expression	Uniprot ID	Annotation	Function
PU262.4	Down	P47043	ZAP1	Zinc ion homeostasis
PU50.217	Down	P53968	CRZ1	Calcium ion homeostasis. Binds to the calcineurin-dependent response element. Transcriptionally regulates PMC1, PMR1, PMR2A, and FKS2.
PU198.24	Up	Q9UTA1	C_2_5B8.19c	DNA-binding transcription repressor activity
PU64.63	Up	Q01981	creA	Controlling carbon source utilization. Represses the transcription of the alcR, alcA, and aldA genes.
PU87.104	Up	Q5EXX3	ZBT38	Transcriptional regulator with bimodal DNA-binding specificity. Binds with a higher affinity to methylated CpG dinucleotides in the consensus sequence
PU70.14	Down	P33749	MSN4	Positive transcriptional factor that acts as a component of the stress responsive system.
PU245.15	Down	Q9BRR0	Zinc finger protein with KRAB and SCAN domains	Specifically represses expression of genes involved in autophagy and lysosome biogenesis/function
PU74.208	Down	P47043	ZAP1	Zinc ion homeostasis
PU305.40	Up	Q9Y2X9	Zinc finger protein 281	Transcription repressor that plays a role in regulation of embryonic stem cells (ESCs) differentiation.
PU5.71	Up	Q9UTA1	C_2_5B8.19c	DNA-binding transcription repressor activity
PU50.75	Down	P39959	YER130C	Unknown
PU193.478	Down	Q9YIB7	ZIC 2-B	Transcriptional repressor that inhibits neurogenesis and induces neural and neural crest differentiation.
PU70.16	own	O74252	steA	Transcription factor involved in sexual reproduction.

Expression: The transcript levels of genes between sclerotia and mycelium.

### 3.2. *PuCRZ1* encodes a nuclear-localized protein

**FIGURE 2:**
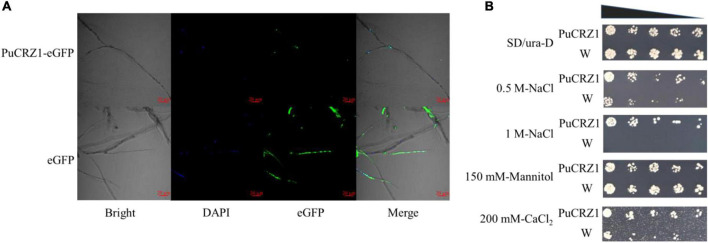
Sublocation and function of *PuCRZ1*. **(A)** Subcellular localization of *PuCRZ1* in *P. umbellatus* mycelia. *PuCRZ1*-eGFP fusion proteins localized to the nucleus in *P. umbellatus* mycelia. Nuclear marker is DAPI. Bar = 20 μm. **(B)** *PuCRZ1* gene could reduce the sensitivity of NaCl and CaCl_2_ in yeast cells. *S. cerevisiae* was transformed with either an empty pYES2 (W) or the pYES2 carrying the cDNA of *PuCRZ1* (PuCRZ1). After obtaining the transformed yeast strains, they were cultured in SD/-Ura liquid medium to OD_600_ = 1.0, and then diluted 100, 1000, 2000, 5000, 10000 times with SD/-Ura liquid medium with D-galactose.

Transcription factors normally perform transcriptional regulatory functions in the nucleus. The coding sequence of *PuCRZ1* was fused in-frame with *eGFP* and transiently expressed in *P. umbellatus* fresh mycelium using *Agrobacterium*-mediated transformation. We observed that free *eGFP* was localized to both the cytoplasm and nucleus of mycelium (Figure 2A), while the nuclear marker (DAPI) and *PuCRZ1*-*eGFP* were co-localized in the nucleus (Figure 2A). The results showed that *PuCRZ1* is a nuclear-localized protein.

### 3.3. *PuCRZ1* function in yeast

In yeast, the growth of the transgenic *PuCRZ1* yeast was unaffected at low concentration of NaCl (0.5 M), but the growth of the wild-type strain was inhibited. At high concentration of NaCl (1 M), the transgenic yeast still grew normally, but the wild-type strain did not grow at all (Figure 2B). Under 200 mM CaCl_2_ conditions, the growth of the wild-type strain was inhibited, while the growth of the transgenic *PuCRZ1* yeast was almost unaffected (Figure 2B), indicating that *PuCRZ1* gene could enhance the resistance of yeast under NaCl and CaCl_2_ stress. Gene expression levels were verified using qRT-PCR (Supplementary Figure 3), and the transcript levels of *PuCRZ1* were significantly different between the wild-type strains and the transgenic *PuCRZ1* yeast.

### 3.4. Genome-wide identification of *PuCRZ1* binding sites and target genes by DAP-seq

**FIGURE 3:**
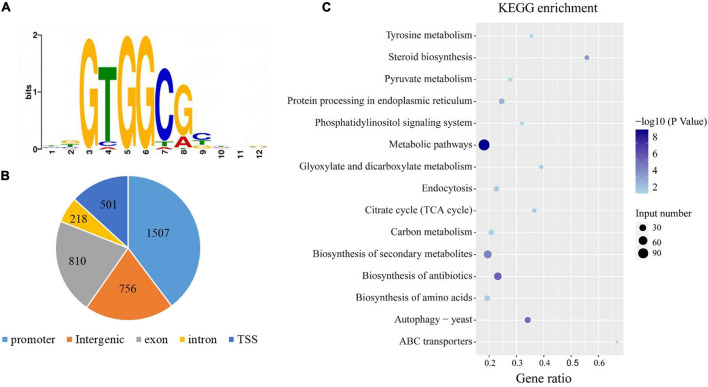
Genome-Wide Identification *PuCRZ1* target genes by DNA affinity purification sequencing. **(A)** *P. umbellatus* DNA motifs bound by *PuCRZ1* protein in DAP-seq. **(B)** Distribution of the *PuCRZ1* binding sites in the *P. umbellatus* genome. **(C)** KEGG annotation of targeted genes bound by *PuCRZ1* protein.

DAP-Seq assay was used to identify the potential target genes that are directly regulated by *PuCRZ1*. Using the Illumina platform, the DAP-Seq assay generated approximately 7 million reads of two biological replicates. Of those reads, approximately 6 million reads were uniquely mapped to the *P. umbellatus* reference genome, an effective mapping ratio of approximately 89.5%. A total of 3792 peaks were significantly associated with GTGGCG motif in the *P. umbellatus* genome for the two biological replicates (Figure 3A). Of all detected peaks, approximately 38% (1507 peaks) were located to the promoter (−2 kb to TSS) (Figure 3B). These 1507 peaks correspond to 1448 genes that were significantly enriched in biosynthesis of antibiotics, biosynthesis of secondary metabolites, steroid biosynthesis, autophagy and endocytosis (Figure 3C).

To further screen for target genes directly regulated by *PuCRZ1*, 168 genes significantly enriched in the top 15 KEGG pathways (*p* < 0.01) were selected for analysis of their expression levels in mycelium and sclerotia, resulting in the identification of 12 genes that may be associated with *P. umbellatus* sclerotia formation (Table 3). Gene expression levels were further verified using qRT-PCR (Supplementary Figure 2), and the transcript levels of three genes (*PuG10*/*PuG11*/*PuG12*) were significantly different between mycelium and sclerotia.

**TABLE 3:** *PuCRZ1* target genes were differentially expressed in sclerotia and mycelia.

No.	ID	Expression	Annotation	Unprot ID
*PuG1*	PU10.189	Up	Multifunctional tryptophan biosynthesis protein	P25170
*PuG2*	PU87.55	Down	Cytochrome P450	O13820
*PuG3*	PU275.193	Up	AMP deaminase	P50998
*PuG4*	PU183.265	Down	Aspartate aminotransferase	P12345
*PuG5*	PU246.38	Down	Cysteine desulfurase, mitochondrial	Q9Y697
*PuG6*	PU193.317	Up	Dihydroorotase	P31301
*PuG7*	PU193.384	Down	Ribonucleoside-diphosphate reductase small chain	Q9C167
*PuG8*	PU198.82	Down	Leukotriene-B4 omega-hydroxylase 3	Q9EP75
*PuG9*	PU201.53	Down	4-coumarate–CoA ligase-like 7	Q9M0X9
*PuG10*	PU64.48	Down	Vacuolar protein sorting-associated protein 45	P97390
*PuG11*	PU159.530	Down	FYVE-domain contain protein	–
*PuG12*	PU294.8	Down	GPI-GlcNAc transferase comlex, PIG-H component domain contain protein	–

Expression: Expression of genes in sclerotia vs. mycelia. The transcript levels of three genes (*PuG10/PuG11/PuG12*) were significantly different between mycelium and sclerotia by qRT-PCR.

BLASTP analysis revealed that *PuG10* is highly homologous to the vacuolar protein sortion-associated protein 45 (uniprot ID: P97390), but no known functional genes of high homology were identified for the other two genes. *PuG11* encodes a FYVE-domain contain protein. The FYVE-domain has been reported to be associated with autophagic degradation (Shen et al., 2020). *PuG12* encodes a GPI-GlcNAc transferase complex, a PIG-H component-domain containing protein that may be involved in the biosynthesis of secondary metabolites (Garneau-Tsodikova et al., 2006).

### 3.5. Y1H assay and EMSA

To determine whether *PuCRZ1* interacts with *PuG10*/*PuG11*/*PuG12*, Y1H assay and EMSA were performed to confirm the binding of *PuCRZ1* to the motif in the promoter region of *PuG10*/*PuG11*/*PuG12*.

**FIGURE 4:**
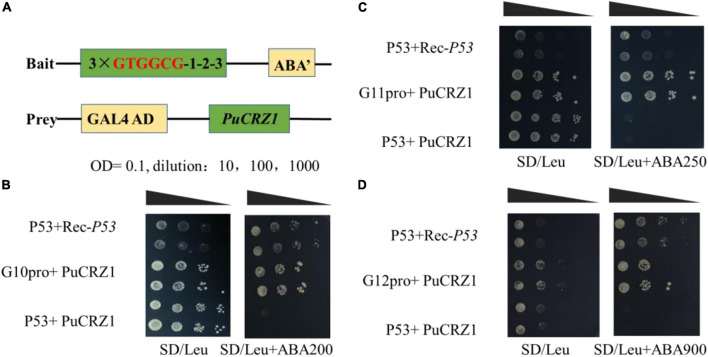
Yeast one-hybrid (Y1H) show *PuCRZ1* binds to the GTGGCG motif in the *PuG10*, *PuG11*, and *PuG12* promoter. **(A)** Plasmid structure of pABAi-promoters and pGADT7- *PuCRZ1*. Three repeats of the motif were used as baits (pAbAi-3 × GTGGCG). **(B)** *PuCRZ1* binds to the *PuG10* promoter. P53+Rec-*P53* represents the positive control, P53+PuCRZ1 represents the negative control. **(C)** *PuCRZ1* binds to the *PuG11* promoter. **(D)** *PuCRZ1* binds to the PuG12 promoter. P53+Rec-P53 represents the positive control, P53+PuCRZ1 represents the negative control. Yeast cells co-expressing pGADT7-PuCRZ1 and the pAbAi-3 × GTGGCG from the *PuG10/PuG11/PuG12* promoter were cultured for 3 days at 30°C in selective medium (SD/-Leu containing 200/250/900 ng/mL AbA). After obtaining the transformed yeast strains, they were cultured in SD/-Leu liquid medium to OD_600_ = 1.0, and then diluted 10^2^, 10^3^, 10^4^, 10^5^ times with 0.9% NaCl. Black triangles represent the dilution ratio of the yeast suspension.

A total of 108 bp tandem copies of *PuG10*, *PuG11*, and *PuG12* promoters (Supplementary Table 2) were fused to the yeast vector (pAbAi) with the reporter gene AUR1-C, respectively (Figure 4A). pAbAi-*PuG10pro* grew on SD/-Ura medium containing 100 ng/mL AbA but was inhibited at 200 ng/mL AbA (Supplementary Figure 4). pAbAi-*PuG11pro* grew on SD/-Ura medium containing 200 ng/mL AbA but was inhibited at 300 ng/mL AbA (Supplementary Figure 4). The pAbAi-*PuG12pro* grew on SD/-Ura medium containing 800 ng/mL AbA but was inhibited at 900 ng/mL AbA (Supplementary Figure 4). The Y1H yeast strain grew on SD/-Leu medium with a plasmid carrying *PuCRZ1* (Figures 4B–D), indicating that *PuCRZ1* can interact with PuG10pro, PuG11pro, and PuG12pro by binding to the GTGGCG element in the upstream regulatory region of the *PuG10*, *PuG11* and *PuG12* promoter.

**FIGURE 5:**
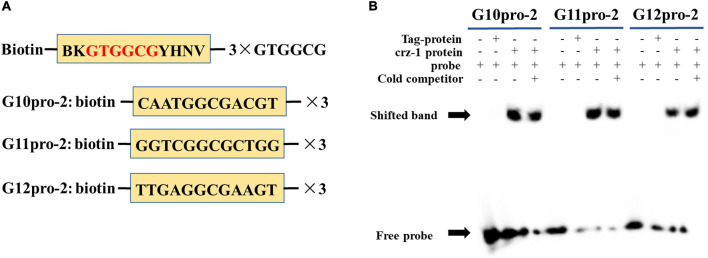
*PuCRZ1* binds to the site in the *PuG10*, *PuG11*, and *PuG12* promoter *in vitro*. **(A)** Schematics of *cis*-elements in EMSA probes. **(B)** Electrophoretic mobility shift assays (EMSA) showing that *PuCRZ1* specifically binds to the GTGGCG-motif. In **(B)** an excess of the cold, unlabeled probe was used as a competitor (lane 4/8/12).

To further clarify the specific binding sites of transcription factor *PuCRZ1* to the promoters of target genes *PuG10*, *PuG11* and *PuG12*, probes were designed at each of the three sites (Figure 5A) for EMSA experiments, and the results showed that *PuCRZ1* could bind to *PuG10*, *PuG11*, and *PuG12* promoters and generate gel shift bands (Figure 5B). *PuCRZ1* binds to the promoters of target gene *PuG10*, *PuG11*, and *PuG12* with specific binding sequences of CAATGGCGACGT, GGTCGGCGCTGG and TTGAGGCGAAGT, respectively.

### 3.6. Functions of *PuCRZ1* and its target genes in *P. umbellatus*

**FIGURE 6:**
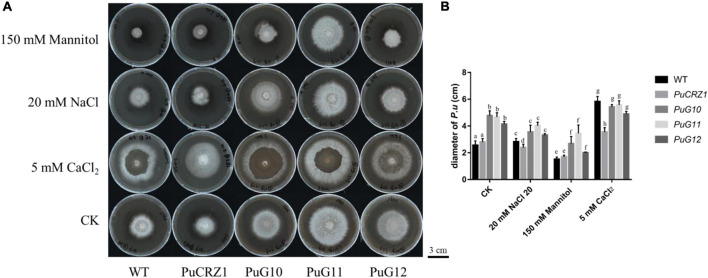
Colony morphology and growth rate of wild-type *P. umbellatus* and transgenic *P. umbellatus* strains (*PuCRZ1/PuG10/PuG11/PuG12*) under different conditions. **(A)** Colony morphology rate of *P. umbellatus* under different conditions. Bar = 3 cm. **(B)** Statistics of mycelial growth rate of different transgenic *P. umbellatus* in different medium. Columns labeled with different letters in the same medium denote a significant difference (*P* < 0.05), *n* = 6.

The transcription factor *PuCRZ1* and its three target genes (*PuG10*, *PuG11*, and *PuG12*) were overexpressed in *P. umbellatus* by *Agrobacterium*-mediated genetic transformation to obtain transgenic strains. The transgenic strains were inoculated onto NaCl (20 mM), mannitol (150 mM), and CaCl_2_ (5 mM) medium, respectively, and the colony growth was observed. After 30 days, the colony diameter of the *PuCRZ1*-overexpressing strain was smaller than those of the wild-type strain in NaCl and CaCl_2_ medium, indicating that *PuCRZ1* overexpression strains were more sensitive to Na^+^ and Ca^2+^. The *PuG10*, *PuG11*, *PuG12* overexpressing strains were significantly larger than the wild-type strains on both untreated medium and mannitol medium, with the *PuG11* overexpressing strain having the largest colony diameter (Figure 6), while there was no difference between the *P. umbellatus* strains grown on NaCl and CaCl_2_ medium (Figure 6). These results suggest that the *PuCRZ1* gene does not directly regulate the tolerance to ion and osmotic stress, but rather responds to stress through its target genes.

## 4. Discussion

This calcineurin-CRZ1 pathway is involved in many developmental functions ranging from the cell wall synthesis, cation homeostasis, lipids metabolism, stress response in eukaryotes (Rusnak and Mertz, 2000). Like calcineurin mutants, CRZ1 deletion mutants in a variety of fungi showed sensitivity to Ca^2+^ and other ionic stresses (Gupta et al., 2022). In addition, Δ*crz1* showed defects in growth, morphology, conidiation, and sclerotium formation in *B*. *cinerea* (Schumacher et al., 2008) and *V. dahlia* (Xiong et al., 2015). *PuCRZ1* is a downstream regulatory target gene of calcineurin. Compared to sclerotia, the expression of *PuCRZ1* is decreased in sclerotium stage. In contrast to our observations, the expression level of *Vdcrz1* is high in precursor of microsclerotia in *V. dahliae* (Xiong et al., 2015). The *PuCRZ1* is associated with sclerotia development in *P. umbellatus*. Sclerotia is the structure of fungi to resist adverse environment, so *PuCRZ1* may also be related to stress response. In our study, *PuCRZ1* gene could reduce the sensitivity of NaCl in yeast cells. Overexpression of the *PuG11*, target gene of *PuCRZ1*, increased the growth rate of *P. umbellatus* under stress (150 mM mannitol). These results indicated that *PuCRZ1* regulated its target genes in response to stress.

In this study, we gained a genome-wide perspective of the direct downstream targets of *PuCRZ1* signaling using a combined approach of DAP-seq and qRT-PCR. CRZ1 in fungi have common as well as unique roles compared to those of their yeast ortholog (Gupta et al., 2022; Yang et al., 2022). As such, we find that the suite of genes regulated by *PuCRZ1* contains a high percentage specific to *P. umbellatus*. Our combined approach identified 1448 direct targets of *PuCRZ1*. The promoter regions of these target genes have specific motifs of GTGGCG. The binding motif of *PuCRZ1* was GTGGCG, which was similar to that of *S. cerevisiae* CRZ1p gene (GNGGC[G/T]CA) (Yoshimoto et al., 2002). Similar sequences are also present in *Aspergillus* genus fungi (GTGGCTC, GAGGCTC) (Spielvogel et al., 2008) and *Candida albicans* (G[C/T]GGT) (Karababa et al., 2006). In *M. oryzae*, CACAGCC, and TTGNTTG have been reported to be *MoCRZ1*-binding motifs (Kim et al., 2010). This suggests that although CRZ1 is conserved across species, its mechanism of action is species specific.

In the study of genes related to the sclerotia development of *P. umbellatus*, we divided the sclerotia development-related genes into seven categories (Bian et al., 2019), including genes related to morphological development, melanin synthesis (polyketide synthase), cell wall synthesis (chitin synthase), defense (WD40) and polysaccharide synthesis (1,3-beta-glucan synthase), according to previous reports. Fungal β-1, 3-glucanases play a key role in cell wall morphogenesis. In our study, 21 genes of these classes were also differentially expressed between sclerotia and mycelia, and three of these genes were regulated by *PuCRZ1* (Supplementary Table 3). The three genes were identified as melanin synthesis gene (PU10.11, annotated as phthiocerol synthesis polyketide synthase type I, PpsA) and morphological development related gene (PU87.55, annotated as Cytochrome P450) genes related to polysaccharide hydrolysis (PU233.5, annotated as 1,3-beta-glucosidase). These results suggested that *PuCRZ1* and its target genes were involved in sclerotia formation and development. According to the DAP-seq results, we also found that *PuCRZ1* was included in the *PuCRZ1* target genes, indicating that the *PuCRZ1* gene also has a regulatory effect on itself.

In our study, three target genes regulated by *PuCRZ1* were found to have the ability to increase mycelial growth rate and cope with mannitol stress. Among them, the protein encoded by *PuG11* gene contains FYVE domain. The FYVE domain is a zinc-finger binding domain that notably occurs in fungi, metazoans, and plant (Gaullier et al., 1998; Shen et al., 2020). Proteins that contain the FYVE zinc-finger domain are recruited to PtdIns3P-containing membranes, participating in numerous biological processes such as membrane trafficking, cytoskeletal regulation, and receptor signaling. The interaction between the FYVE domain and PI3P was found to be very specific (Burd and Emr, 1998). In yeast species, the function of FYVE-containing protein (VPS34), is associated with endocytosis and transcription to the tonoplast, and in deletion mutants of this gene, migration from the Golgi and plasma membrane to the tonoplast is impaired (Vieira et al., 2004). In *Phytophthora sojae*, FYVE-containing protein (PSFP1) plays an important role in fungal vegetative growth and virulence. Knockout of this gene resulted in decreased mycelium growth and pathogenicity, and increased sensitivity to hydrogen peroxide (Zhang et al., 2021). In *Arabidopsis*, FLVY-domain-contain proteins are related to stress tolerance (Pan et al., 2020), protein transport (Kolb et al., 2015), and ABA signaling pathway regulation (Li et al., 2019a). However, the regulatory mechanism on FLVY-domain-contain proteins have not yet been reported. In our study, a target gene of *PuCRZ1* (*PuG11*) was found to have FlVY-domain-contain. It also enhances its growth rate in osmotic stress (mannitol). This study expands the function and mechanism of FLVY-domain contain proteins in fungi. In our study, we found that *PuG11*, a target gene of *PuCRZ1*, encodes a FLVY-domain protein. Overexpression of *PuG11* in mycelia *of P. umbellatus* not only enhanced the growth rate of mycelia, but also enhanced its growth rate under osmotic stress (mannitol). This study enhances our understanding of the function and mechanism of FLVY-domain-containing proteins in fungi.

**FIGURE 7:**
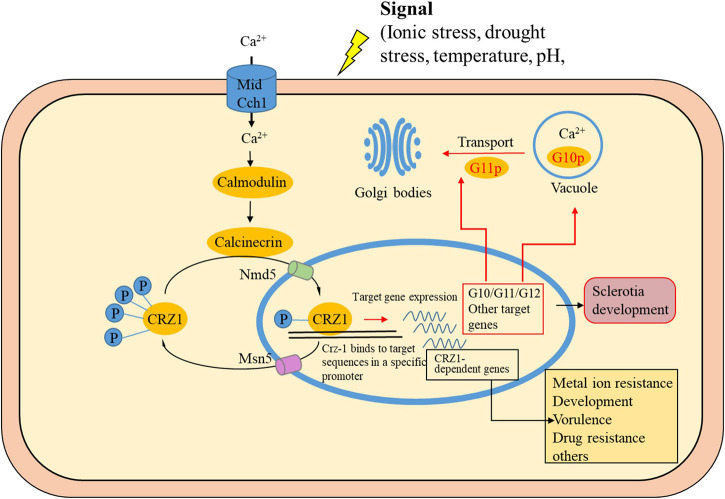
Schematic representation of CRZ1 and target genes in Ca^2+^ pathways. When the cytosolic Ca^2+^ concentration increases, calmodulin activates calcineurin, which in turn dephosphorylates CRZ1. CRZ1 is then imported into the nucleus and induces or represses expression of its target genes. After phosphorylation (P), CRZ1 is exported from the nucleus. The cylinders represent channel proteins, and the yellow ovals represent proteins inside the cell. Genes in the black box are those reported in the literature (Thewes, 2014). Genes in this study are marked in red boxes or red arrows.

This study is the first of its kind where DAP-seq technology has been applied to medicinal fungi. The correlation of comprehensive whole genome expression data with results from DAP-seq have allowed for significant refinement of the predicted targets of *PuCRZ1*. This study reveals conserved elements of the calcium/calcineurin signaling pathway and allow for responses tailored to biology of the organism. Calcium signaling is a ubiquitous and complicated aspect of cell physiology. This study represents a major advance in our understanding of this pathway in *P. umbellatus* and provides the launching point for the functional characterization of the genes and interactions it implicates. Figure 7 depicts our proposed model resulting from this work and includes our new findings of predictive roles for *PuCRZ1* in mycelium growth, feedback regulation, and osmotic stress response. Future research should focus on the function of other transcription factors in *P. umbellatus* sclerotia formation and decipher their regulatory pathways. Our results will enrich our understanding of the CRZ1 and FYVE domain-containing protein and fill the international gap in the research on the functions of CRZ1 and FYVE domain-containing protein in *P. umbellatus*.

## Data availability statement

The data presented in the study are deposited in the National Genomics Data Center (NGDC) with project number: PRJCA008746 and the *Polyporus umbellatus* genome accession number: GWHBQTP00000000 https://ngdc.cncb.ac.cn/bioproject/browse/PRJCA008746.

## Author contributions

YY designed the experiments. PH performed the experiments and wrote the original draft manuscript. PH and ZH analyzed the data. YZ, YY, and LH supervised the study and revised the manuscript. All authors have read and agreed to the published version of the manuscript.

## References

[B1] Bandara A. R. Rapior S. Bhat D. J. Kakumyan P. Chamyuang S. Xu J. (2015). *Polyporus umbellatus*, an edible-medicinal cultivated mushroom with multiple developed health-care products as food, medicine and cosmetics: a review. *Cryptogamie Mycologie* 36 3–42.

[B2] Bartlett A. O’Malley R. C. Huang S. C. Galli M. Nery J. R. Gallavotti A. (2016). Mapping genome-wide transcription-factor binding sites using DAP-seq. *Nat. Protoc.* 12 1659–1672. 10.1038/nprot.2017.055 28726847 PMC5576341

[B3] Berridge M. J. Bootman M. D. Roderick H. L. (2003). Calcium signalling: dynamics, homeostasis and remodelling. *Nat. Rev. Mol. Cell Biol.* 4 517–529. 12838335 10.1038/nrm1155

[B4] Bian X. Y. Pei T. L. Liang Z. S. Chang Z. Y. (2019). A landscape of transcriptome analysis of three sclerotia growth stages in *Polyporus umbellatus*. *China J. Chin. Materia Medica* 44 3718–3723. 10.19540/j.cnki.cjcmm.20190701.112 31602944

[B5] Burd C. G. Emr S. D. (1998). Phosphatidylinositol(3)-phosphate signaling mediated by specific binding to RING FYVE domains. *Mol. Cell* 2 157–162. 10.1016/s1097-2765(00)80125-2 9702203

[B6] Chen L. Tong Q. Zhang C. Ding K. (2019). The transcription factor FgCrz1A is essential for fungal development, virulence, deoxynivalenol biosynthesis and stress responses in *Fusarium graminearum*. *Curr. Genet.* 665 153–166. 10.1007/s00294-018-0853-5 29947970

[B7] Chen X. Liu Y. Keyhani N. O. Xia Y. Cao Y. (2017). The regulatory role of the transcription factor Crz1 in stress tolerance, pathogenicity, and its target gene expression in *Metarhizium acridum*. *Appl. Microbiol. Biotechnol.* 101 5033–5043. 10.1007/s00253-017-8290-9 28424845

[B8] Chinese Pharmacopoeia Commission (2020). *Pharmacopoeia of the People’ s Republic of China.* Beijing: China Medical Science Press.

[B9] Garneau-Tsodikova S. Dorrestein P. C. Kelleher N. L. Walsh C. T. (2006). Protein assembly line components in prodigiosin biosynthesis: characterization of PigA,G,H,I,J. *J. Am. Chem. Soc.* 128 12600–12601. 10.1021/ja063611l 17002325

[B10] Gaullier J. M. Simonsen A. D’Arrigo A. Bremnes B. Stenmark H. Aasland R. (1998). FYVE fingers bind PtdIns(3)P. *Nature* 394 432–433. 9697764 10.1038/28767

[B11] Gupta S. Kumar A. Tamuli R. (2022). CRZ1 transcription factor is involved in cell survival, stress tolerance, and virulence in fungi. *J. Biosci.* 47:66. 36408540

[B12] He F. Zhang X. Mafurah J. J. Zhang M. Qian G. Wang R. (2016). The transcription factor VpCRZ1 is required for fruiting body formation and pathogenicity in *Valsa pyri*. *Microb. Pathog.* 95 101–110. 10.1016/j.micpath.2016.02.018 26970115

[B13] Heinz S. Benner C. Spann N. Bertolino E. Lin Y. C. Laslo P. (2010). Simple combinations of lineage-determining transcription factors prime cis-regulatory elements required for macrophage and B cell identities. *Mol. Cell* 38 576–589. 10.1016/j.molcel.2010.05.004 20513432 PMC2898526

[B14] Ji X. Wang L. Nie X. He L. Zang D. Liu Y. (2014). A novel method to identify the DNA motifs recognized by a defined transcription factor. *Plant Mol. Biol. Rep.* 86 367–380. 10.1007/s11103-014-0234-5 25108460

[B15] Karababa M. Valentino E. Pardini G. Coste A. T. Bille J. Sanglard D. (2006). CRZ1, a target of the calcineurin pathway in *Candida albicans*. *Mol. Microbiol.* 59 1429–1451. 10.1111/j.1365-2958.2005.05037.x 16468987

[B16] Kim S. Hu J. Oh Y. Park J. Choi J. Lee Y. H. (2010). Combining ChIP-chip and expression profiling to model the MoCRZ1 mediated circuit for Ca/calcineurin signaling in the rice blast fungus. *PLoS Pathog.* 6:e1000909. 10.1371/journal.ppat.1000909 20502632 PMC2873923

[B17] Kolb C. Nagel M. K. Kalinowska K. Hagmann J. Ichikawa M. Anzenberger F. (2015). FYVE1 is essential for vacuole biogenesis and intracellular trafficking in *Arabidopsis*. *Plant Physiol.* 167 1361–1373. 10.1104/pp.114.253377 25699591 PMC4378156

[B18] Langmead B. Salzberg S. L. (2012). Fast gapped-read alignment with Bowtie 2. *Nat. Methods* 9 357–359. 10.1038/nmeth.1923 22388286 PMC3322381

[B19] Li H. Li Y. Zhao Q. Li T. Wei J. Li B. (2019a). The plant ESCRT component FREE1 shuttles to the nucleus to attenuate abscisic acid signalling. *Nat. Plants* 5 512–524. 30962512 10.1038/s41477-019-0400-5

[B20] Li H. Yan Z. Xiong Q. Chen X. Lin Y. Xu Y. (2019b). Renoprotective effect and mechanism of polysaccharide from *Polyporus umbellatus* sclerotia on renal fibrosis. *Carbohydr. Polym.* 212 1–10. 10.1016/j.carbpol.2019.02.026 30832835

[B21] Li H. Zhong J. J. (2020). Role of calcineurin-responsive transcription factor CRZ1 in ganoderic acid biosynthesis by *Ganoderma lucidum*. *Process Biochem.* 95 166–173.

[B22] Liu G. K. Yang T. X. Wang J. R. (2021). Polysaccharides from *Polyporus umbellatus*: a review on their extraction, modification, structure, and bioactivities. *Int. J. Biol. Macromol.* 189 124–134. 10.1016/j.ijbiomac.2021.08.101 34419536

[B23] Liu Q. Ma H. Zhang Y. Dong C. (2018). Artificial cultivation of true morels: current state, issues and perspectives. *Crit. Rev. Biotechnol.* 38 259–271. 10.1080/07388551.2017.1333082 28585444

[B24] Machanick P. Bailey T. L. (2011). MEME-ChIP: motif analysis of large DNA datasets. *Bioinformatics* 27 1696–1697. 10.1093/bioinformatics/btr189 21486936 PMC3106185

[B25] O’Malley R. C. Huang S. C. Song L. Lewsey M. G. Bartlett A. Nery J. R. (2016). Cistrome and epicistrome features shape the regulatory DNA landscape. *Cell* 165 1280–1292. 27203113 10.1016/j.cell.2016.04.038 PMC4907330

[B26] Pan W. Zheng P. Zhang C. Wang W. Li Y. Fan T. (2020). The effect of ABRE BINDING FACTOR 4-mediated FYVE1 on salt stress tolerance in *Arabidopsis*. *Plant Sci.* 296:110489. 10.1016/j.plantsci.2020.110489 32540007

[B27] Quinlan A. R. Hall I. M. (2010). BEDTools: a flexible suite of utilities for comparing genomic features. *Bioinformatics* 26 841–842. 10.1093/bioinformatics/btq033 20110278 PMC2832824

[B28] Rusnak F. Mertz P. (2000). Calcineurin: form and function. *Physiol. Rev.* 80 1483–1521. 11015619 10.1152/physrev.2000.80.4.1483

[B29] Schumacher J. de Larrinoa I. F. Tudzynski B. (2008). Calcineurin-responsive zinc finger transcription factor CRZ1 of *Botrytis cinerea* is required for growth, development, and full virulence on bean plants. *Eukaryotic Cell* 7 584–601. 10.1128/EC.00426-07 18263765 PMC2292616

[B30] Shen W. Wei J. Gao C. (2020). Functional analysis of plant FYVE domain proteins in endosomal trafficking. *Methods Mol. Biol.* 2177 83–94. 10.1007/978-1-0716-0767-1_8 32632807

[B31] Smith M. E. Henkel T. W. Rollins J. A. (2015). How many fungi make sclerotia? *Fungal Ecol.* 13 211–220.

[B32] Soriani F. M. Malavazi I. da Silva Ferreira M. E. Savoldi M. Von Zeska Kress M. R. de Souza Goldman M. H. (2008). Functional characterization of the *Aspergillus fumigatus* CRZ1 homologue, CrzA. *Mol. Microbiol.* 67 1274–1291. 10.1111/j.1365-2958.2008.06122.x 18298443

[B33] Spielvogel A. Findon H. Arst H. N. Araújo-Bazán L. Hernández-Ortíz P. Stahl U. (2008). Two zinc finger transcription factors, CrzA and SltA, are involved in cation homoeostasis and detoxification in *Aspergillus nidulans*. *Biochem. J.* 414 419–429. 10.1042/BJ20080344 18471095

[B34] Stathopoulos A. M. Cyert M. S. (1997). Calcineurin acts through the CRZ1/TCN1-encoded transcription factor to regulate gene expression in yeast. *Genes Dev.* 11 3432–3444. 10.1101/gad.11.24.3432 9407035 PMC316814

[B35] Thewes S. (2014). Calcineurin-Crz1 signaling in lower eukaryotes. *Eukaryot Cell* 13 694–705. 24681686 10.1128/EC.00038-14 PMC4054267

[B36] Vieira O. V. Harrison R. E. Scott C. C. Stenmark H. Alexander D. Liu J. (2004). Acquisition of Hrs, an essential component of phagosomal maturation, is impaired by mycobacteria. *Mol. Cell. Biol.* 24 4593–4604. 10.1128/MCB.24.10.4593-4604.2004 15121875 PMC400451

[B37] Xing Y. M. Li B. Zeng X. Zhou L. S. Lee T. S. Lee M. W. (2021). Use of transcriptomic profiling to identify candidate genes involved in *Polyporus umbellatus* sclerotial formation affected by oxalic acid. *Sci. Rep.* 11:17326. 10.1038/s41598-021-96740-7 34462479 PMC8405643

[B38] Xing Y. M. Yin W. Q. Liu M. M. Wang C. L. Guo S. X. (2015). Oxalic acid and sclerotial differentiation of *Polyporus umbellatus*. *Sci. Rep.* 5:10759. 10.1038/srep10759 26030006 PMC5377064

[B39] Xing Y. M. Zhang L. C. Liang H. Q. Lv J. Song C. Guo S. X. (2013). Sclerotial formation of *Polyporus umbellatus* by low temperature treatment under artificial conditions. *PLoS One* 8:e56190. 10.1371/journal.pone.0056190 23437090 PMC3577777

[B40] Xiong D. Wang Y. Tang C. Fang Y. Zou J. Tian C. (2015). VdCrz1 is involved in microsclerotia formation and required for full virulence in *Verticillium dahliae*. *Fungal Genet. Biol.* 82 201–212. 10.1016/j.fgb.2015.07.011 26235044

[B41] Yang Y. Xie P. Li Y. Bi Y. Prusky D. B. (2022). Updating insights into the regulatory mechanisms of calcineurin-activated transcription factor Crz1 in pathogenic fungi. *J. Fungi* 8:1082. 10.3390/jof8101082 36294647 PMC9604740

[B42] Yao J. Shen Z. Zhang Y. Wu X. Wang J. Sa G. (2020). *Populus euphratica* WRKY1 binds the promoter of H+-ATPase gene to enhance gene expression and salt tolerance. *J. Exp. Bot.* 71 1527–1539. 10.1093/jxb/erz493 31680166 PMC7031066

[B43] Yoshimoto H. Saltsman K. Gasch A. P. Li H. X. Ogawa N. Botstein D. (2002). Genome-wide analysis of gene expression regulated by the calcineurin/Crz1p signaling pathway in *Saccharomyces cerevisiae*. *J. Biol. Chem.* 277 31079–31088. 10.1074/jbc.M202718200 12058033

[B44] Zhang H. Zhao Q. Liu K. Zhang Z. Wang Y. Zheng X. (2009). MgCRZ1, a transcription factor of *Magnaporthe grisea*, controls growth, development and is involved in full virulence. *FEMS Microbiol. Lett.* 293 160–169. 10.1111/j.1574-6968.2009.01524.x 19260966

[B45] Zhang J. Du X. Zhou X. Jin D. Miao J. Liu X. (2021). An FYVE-domain-containing protein, PsFP1, is involved in vegetative growth, oxidative stress response and virulence of *Phytophthora sojae*. *Int. J. Mol. Sci.* 22:6601. 10.3390/ijms22126601 34202990 PMC8233823

[B46] Zhang J. Silao F. G. Bigol U. G. Bungay A. A. Nicolas M. G. Heitman J. (2012). Calcineurin is required for pseudohyphal growth, virulence, and drug resistance in *Candida lusitaniae*. *PLoS One* 7:e44192. 10.1371/journal.pone.0044192 22952924 PMC3432075

[B47] Zhang Y. Liu T. Meyer C. A. Eeckhoute J. Johnson D. S. Bernstein B. E. (2008). Model-based analysis of ChIP-Seq (MACS). *Genome Biol.* 9:R137. 10.1186/gb-2008-9-9-r137 PMC2592715 18798982

[B48] Zheng H. Jing L. Jiang X. Pu C. Zhao S. Yang J. (2021). The ERF-VII transcription factor SmERF73 coordinately regulates tanshinone biosynthesis in response to stress elicitors in Salvia miltiorrhiza. *New Phytol.* 231 1940–1955. 10.1111/nph.17463 33983629

