# Supplementary material for: PuCRZ1, an C2H2 transcription factor from Polyporus umbellatus, positively regulates mycelium response to osmotic stress
Source: Front Microbiol. 2023 Apr 6;14:1131605. doi: 10.3389/fmicb.2023.1131605 (PMC10115967; doi:10.3389/fmicb.2023.1131605)
Supplement: Supplementary file 1 [file Data_Sheet_1.PDF]

Supplementary Material

1 Supplementary Figures

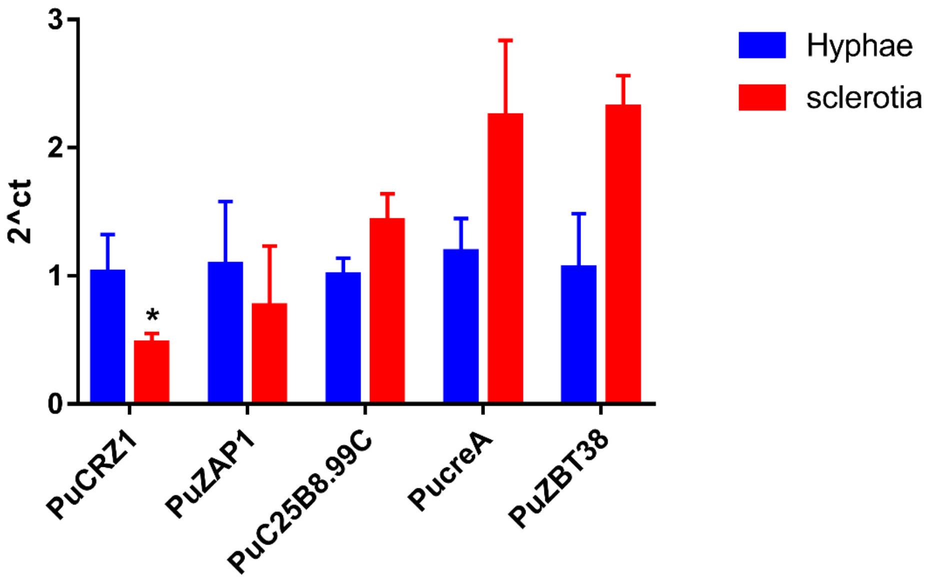

Supplementary Figure 1 qRT-PCR results for 5 genes of C2H2 gene famliy in *P. umbellatus* between hyphae and sclerotium. * *P*<0.05

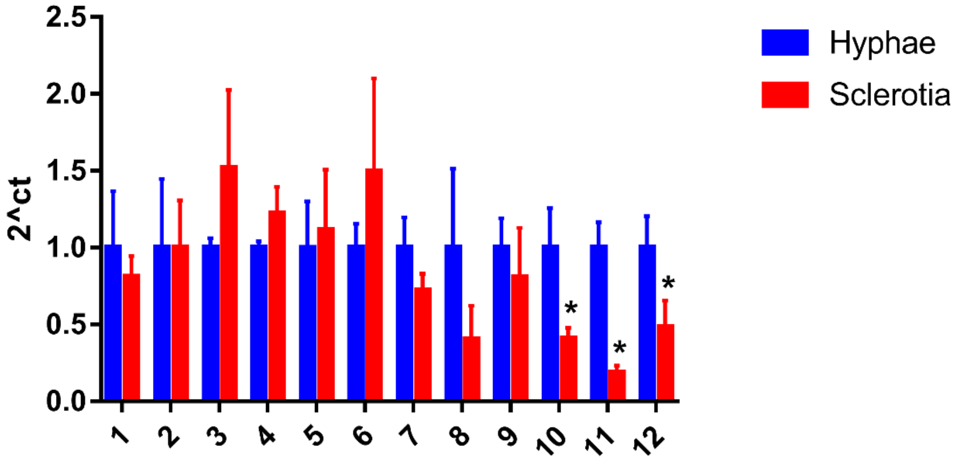

Supplementary Figure 2 qRT-PCR results for 12 target genes of *PuCRZ1* between mycelium and sclerotium. 1-12 is for *PuG1* to *PuG12* in Table 2. The transcript levels of three genes (*PuG10*/*PuG11*/*PuG12*) were significantly different between hyphae and sclerotia.

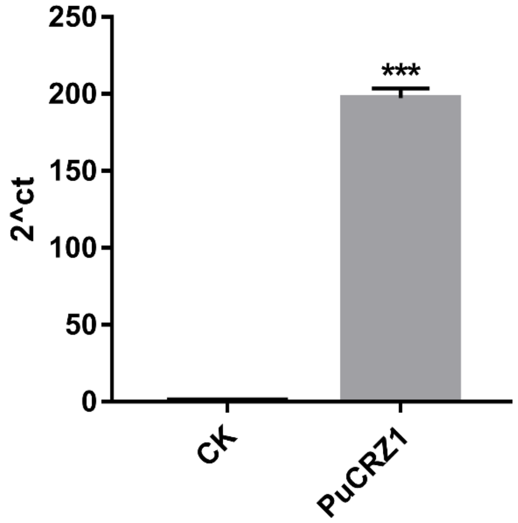

Supplementary Figure 3 qRT-PCR results for PuCRZ1 in yeast. The transcript levels of *PuCRZ1* were significantly different between the wild-type strains and the transgenic *PuCRZ1* yeast.

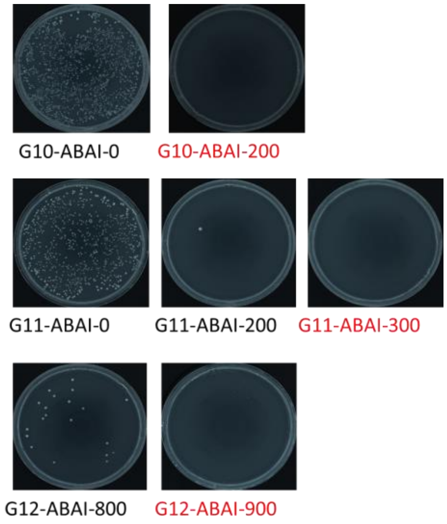

Supplementary Figure 4 Screening for ABA concentration in Y1H

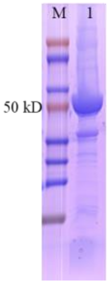

Supplementary Figure 5 SDS-PAGE result of purified PuCRZ1 protein

2 Supplementary Tables

Supplementary Table 1 Primers in study

Name	Sequence
**RT-PCR primers**	
PuG1-F	GAAGGTGATTGGGGTGAACAAC
PuG1-R	TTATACACCCTCACATCCTCTGG
PuG2-F	CAGAACCCCAAGAACTATCTCGT
PuG2-R	TTACGACATGATCCCACTCCATC
PuG3-F	CGCATATATCCTCACCCTGACAA
PuG3-R	GAGTATAAACGACCACTTGTGCG
PuG4-F	GCTGAGAAGGCCCATATCTACAT
PuG4-R	CATGCCTCAAATCTTAGAGCTCG
PuG5-F	CATCATCACGACTCAGACTGAAC
PuG5-R	CTGATAACGCCGTTCTTCTGAAC
PuG6-F	GTCACAAAGCATGTTCGTCTAGG
PuG6-R	CCTCCAGCTGTGACTTGTATAGT
PuG7-F	TTATGGACATGATCTCGCTCCAG
PuG7-R	CAGAAGTCCTCGTCGATACAGAA
PuG8-F	AGTGTAGATGTCTTCTCCGCATC
PuG8-R	GGATGTAGAAGAGAGCAGCAAGA
PuG9-F	TTCCTTCTCTTCTCGAGTGGAAC
PuG9-R	GCTTATTGACGGACTGCTTCATC
28S rDNA-F	GAGTCGAGTTGTTTGGGAATGC
28S rDNA-R	TCTCTTTCCAAAGTTCTTTTCATCTTT

Supplementary Table 2 Probe in Y1H and EMSA

Name	**Sequence**	**Note**
Comp-biotin	CGGTGGCTGTGCCGGTGGCTGTGCCGGTGGCTGTGC	
Comp-biotin-R	GCACAGCCACCGGCACAGCCACCGGCACAGCCACCG	
G10-biotin-2	CaatggcgacgtCaatggcgacgtCaatggcgacgt	5'-biotin
G10-biotin-2-R	acgtcgccattGacgtcgccattGacgtcgccattG	5'-biotin
G11-biotin-2	GgtcggcgctggGgtcggcgctggGgtcggcgctgg	5'-biotin
G11-biotin-2-R	ccagcgccgacCccagcgccgacCccagcgccgacC	5'-biotin
G12-biotin-2	gggaggcgagtggggaggcgagtggggaggcgagtg	5'-biotin
G12-biotin-2-R	cactcgcctccccactcgcctccccactcgcctccc	5'-biotin
PuG10 promoters	AagaggcgtatcAagaggcgtatcAagaggcgtatcCaatggcgacgtCaatggc gacgtCaatggcgacgtGgtaggcgagctGgtaggcgagctGgtaggcgagct	
PuG11 promoters	GtgaggcggcgaGtgaggcggcgaGtgaggcggcgaGgtcggcgctggGgtcg gcgctggGgtcggcgctggTcaaggcgcggtTcaaggcgcggtTcaaggcgcggt	
PuG12 promoters	ttgaggcgaagtttgaggcgaagtttgaggcgaagtgggaggcgagtggggaggcga gtggggaggcgagtgcggaggcgcgttcggaggcgcgttcggaggcgcgtt	

Supplementary Table 3 Genes related to the sclerotia development of *P. umbelleaus*

number	name	*PuCRZ1* target gene	unprot ID	function
**Major facilitator superfamily**				
1	PU105.4	No	O94343	Uncharacterized MFS-type transporter C1271.10c
2	PU159.93	No	Q10084	Uncharacterized transporter mfs2
3	PU187.18	No	O94343	Uncharacterized MFS-type transporter C1271.10c
4	PU50.112	No	O32182	Uncharacterized MFS-type transporter YusP
5	PU6.18	No	Q7Z9I0	Uncharacterized MFS-type transporter C409.08
**cell wall synthesis**				
6	PU201.13	No	Q4P9K9	Chitin synthase 8
**melanin synthesis**				
7	PU10.11	Yes	P9WQE6	Phthiocerol synthesis polyketide synthase type I PpsA
8	PU193.338	No	P37693	Polyketide synthase HetM
**morphological development**				
9	PU318.1	No	Q9VL92	Cytochrome P450 4e3
10	PU87.55	Yes	O13820	Cytochrome P450 61
11	PU43.60	No	Q12609	Probable sterigmatocystin biosynthesis P450 monooxygenase stcF
**Gsase**				
12	PU62.267	No	Q9C3Y4	GTP-binding protein rhoA
**Defense**				
13	PU159.197	No	P87053	F-box/WD repeat-containing protein pof1
14	PU296.74	No	Q09855	F-box/WD repeat-containing protein pof11
**Polysaccharide synthesis**				
15	PU172.36	No	O13716	Glucan endo-1,3-alpha-glucosidase agn1
16	PU172.46	No	Q8N0N3	Beta-1,3-glucan-binding protein
17	PU193.451	No	O13941	Uncharacterized beta-glucan synthesis-associated protein
18	PU233.5	Yes	O74799	Glucan 1,3-beta-glucosidase
19	PU275.188	No	Q7M4T0	Endo-1,6-beta-D-glucanase
20	PU6.179	No	Q8N0N3	Beta-1,3-glucan-binding protein
21	PU62.33	No	O74799	Glucan 1,3-beta-glucosidase
